# Cutinase ACut2 from *Blastobotrys* *raffinosifermentans* for the Selective Desymmetrization of the Symmetric Diester Diethyl Adipate to the Monoester Monoethyl Adipate

**DOI:** 10.3390/microorganisms10071316

**Published:** 2022-06-29

**Authors:** Marion Rauter, Daniela Nietz, Gotthard Kunze

**Affiliations:** 1 Orgentis Chemicals GmbH, Bahnhofstr. 3–5, Gatersleben, D-06466 Stadt Seeland, Germany; mar.rauter@orgentis.com; 2 Leibniz Institute of Plant Genetics and Crop Plant Research (IPK), Corrensstr. 3, Gatersleben, D-06466 Stadt Seeland, Germany

**Keywords:** diethyl adipate, monoethyl adipate, cutinase 2 (ACut2), desymmetrization, *Blastobotrys raffinosifermentans*

## Abstract

Monoethyl adipate (MEA) is a highly valuable monoester for activating resistance mechanisms and improving protective effects in pathogen-attacked plants. The cutinase ACut2 from the non-conventional yeast *Blastobotrys (Arxula) raffinosifermentans (adeninivorans)* was used for its synthesis by the desymmetrization of dicarboxylic acid diester diethyl adipate (DEA). Up to 78% MEA with 19% diacid adipic acid (AA) as by-product could be synthesized by the unpurified ACut2 culture supernatant from the *B. raffinosifermentans* overexpression strain. By adjusting pH and enzyme concentration, the selectivity of the free ACut2 culture supernatant was increased, yielding 95% MEA with 5% AA. Selectivity of the carrier immobilized ACut2 culture supernatant was also improved by pH adjustment during immobilization, as well as carrier enzyme loading, ultimately yielding 93% MEA with an even lower AA concentration of 3–4%. Thus, optimizations enabled the selective hydrolysis of DEA into MEA with only a minor AA impurity. In the up-scaling, a maximum of 98% chemical and 87.8% isolated MEA yield were obtained by the adsorbed enzyme preparation with a space time yield of 2.6 g L^−1^ h^−1^. The high monoester yields establish the ACut2-catalyzed biosynthesis as an alternative to existing methods.

## 1. Introduction

**Figure 1:**
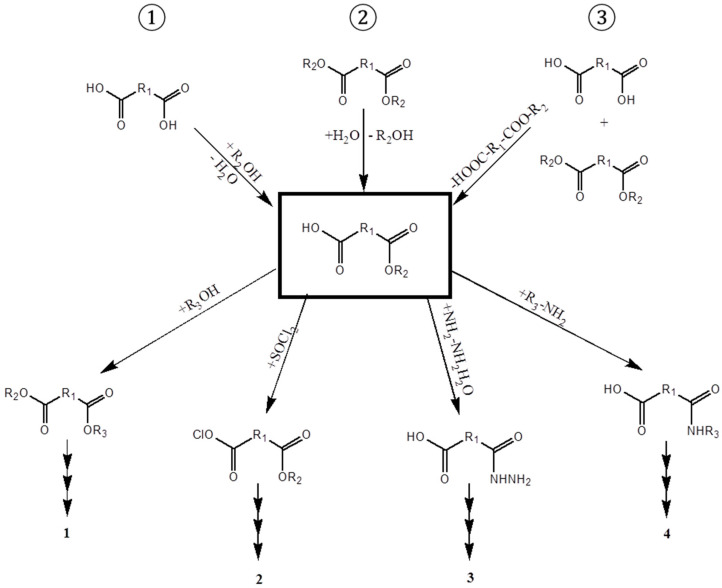
Synthesis of aliphatic monoesters by esterification, selective monohydrolysis, and transesterification, and the following reactions for which monoesters are used [7,8,9,10,11,12,13]. 1, 2, 3 and 4 are described in Table 1.

Monoesters from symmetric dicarboxylic acids or diols possess two different functional groups—on the one side an ester group, and on the other side a carboxylate or an alcohol residue—offering different reactivities (Figure 1, middle). This makes them interesting as highly versatile building blocks, e.g., for anti-cancer and anti-tumor agents [1,2], as plasticizers [3], or as educts for further chemicals [4] (Figure 1, lower range, and Table 1). There is an increasing demand for these monoesters as monomeric linkers or spacer groups, especially in medicinal chemistry and for dental applications [5,6].

For oligonucleotide synthesis, dicarboxylate units are spacers between a phosphoramidite group bonded to a solid support and the first covalently attached nucleoside [18]. In that way, the spacer provides flexibility and accessibility of functional groups, and serves as a distance piece from the polymer. The length and the design of the spacer arms decide the chemical, physical and biological characteristics, as well as the flexibility and formability [19,20,21,22]. The introduction of both functionalities can be achieved by using those monoesters. They are linked with the polymer, as well as their functional groups, e.g., by the nucleophilic substitution with hydroxyl- or carboxy-groups; in doing so, leaving groups such as methylsulfonyl- or methoxy-groups to be removed from the polymer [23].

Monoesters can be obtained by ① esterification of a dicarboxylic acid and an alcohol or a diol and a carboxylic acid, ② hydrolysis of the diester, or ③ transesterification of the diester with an alcohol for dicarboxylic acid esters or with a carboxylic acid for diolesters (Figure 1, upper range). These reactions are non-selective for chemical synthesis, so that the formed monoester is further hydrolyzed to diacid or diol. Monoester synthesis by anhydride esterification needs high temperatures and a huge excess of the alcohol, which is neither eco-friendly, nor sustainable.

On the contrary, enzymatic reactions can be selective; if the rate constant for the diester hydrolysis is higher compared to that for the monoester hydrolysis, then high monoester concentrations can be obtained. Compared to the chemical synthesis, lower temperatures and alcohol concentrations have to be used.

Some lipases and pig liver esterase have already been successfully used for the monohydrolysis of various diesters [24,25,26,27,28,29,30]. Under the conditions tested, high monoester yields of 96 to 99+% were obtained [24,26,27]. It is no foregone conclusion that lipases/esterases carry out chemoselective hydrolysis of only one of the two ester functions of the diester compound. CALB was not chemoselective at all, despite various adaptations of the reaction conditions [28]. Bianchi et al. [31] used two lipases for the hydrolysis of terephthalic acid diesters, gaining only 60% monoester and 40% diacid. 

In some cases, optimizing the reaction conditions can overcome the selectivity problems. Along these lines, Ehlert and coworkers [28] improved the chemoselectivity for four lipases until no diacid was detectable by adjusting the reaction time, temperature, and organic solvent concentration. The conversion, however, was low (max. 22%) [28]. By employing cell lysates of *Brevibacterium imperiale* B222 instead of whole cells, a more chemoselective hydrolysis of terephthalic acid diester substrate could be achieved [31]. As a result, the diacid concentration was lowered from 12 to only 2% [31]. The increased chemoselectivity was explained by assuming that the intact cells contain two esterases acting on the same substrate, with the one with the lesser chemoselectivity being more sensitive to the lysis procedure [31].

The vast majority of studies report on the lipase-catalyzed desymmetrizations of cyclic or aromatic diesters [24,26,31,32,33]. Nietz et al. describe the aliphatic diester hydrolysis of diethyl adipate (DEA) by purified intracellular lipase Alip2-c6hp expressed in *Blastobotrys raffinosifermentans*. This reaction produced 96% monoethyl adipate (MEA), and only 1.6% of the unwanted side-product adipic acid (AA) [27]. Other aliphatic dicarboxylic acid esters were completely hydrolyzed to their corresponding monoesters [27]. Aliphatic monoester as MEA and derivatives thereof have positive effects on pathogen attacked plants by activating resistance mechanisms, thus improving protective effects [34,35,36,37]. Here we focus on the desymmetrization of the aliphatic diester DEA to the aliphatic monoester MEA by the cutinase ACut2-6hp from the non-conventional yeast *B. raffinosifermentans.* This, to our knowledge, is the first time a cutinase has been used for the desymmetrization of a diester. Cutinases belong to the serine hydrolases and are specific for primary alcohol esters [38]. They hydrolyze the polyesters cutin and suberin composed of hydroxyl and epoxy fatty acids, as well as further industrially relevant compounds [39]. Three cutinases named ACut1, ACut2, and ACut3 were already identified in the yeast *B. raffinosifermentans*, and the corresponding genes have been overexpressed in the yeast itself [40]. All three biocatalysts have quite similar biochemical characteristics and substrate spectra. Fed-batch cultivation gave the highest activities for the ACut2-6hp-producing strain [40], which was thus used for the experiments described in the present paper.

The culture supernatant of a ACut2-6hp overexpression strain was used without any purification step. Although in initial experiments DEA hydrolysis also produced AA, subsequent optimizations improved the chemoselectivity of the biocatalyst, gaining a high conversion and a high monoester concentration. For industrial application, supernatant immobilization on carriers was tested and optimized concerning monoester formation, and the final reactions for DEA hydrolysis were up-scaled. Without the need for enzyme purification steps, and due to the longevity of the enzyme immobilizates, this work confirms the potential of ACut2-6hp as a biocatalyst for chemoselective monoester synthesis from aliphatic diesters with different chain lengths.

## 2. Materials and Methods

### 2.1. Chemicals

Chemicals were purchased as follows: DEA, MEA, and AA were from Alfa Aesar (Kandel, Germany). 2-Nonanone was from Acros (Geel, Belgium), and acetophenone and para-nitrophenyl (pNP)-butyrate were from Sigma (Steinheim, Germany).

### 2.2. Production of ACut2-6hp Enzyme Preparations

*B. raffinosifermentans* strain G1212/YRC102-ACut2-6H derived from the wild type strain *B. raffinosifermentans* LS3 [41] was used for the synthesis of recombinant ACut2-6hp [40]. Shake flask cultivation was done in 200 mL flasks in yeast minimal medium supplemented with 20 g L^−1^ glucose as a carbon source and 43 mM NaNO_3_ (YMM-glucose-NaNO_3_) as nitrogen sources or YEPD (Sigma, Steinheim, Germany) at 30 °C for 48 h, 180 rpm. Cells were removed by centrifugation (5 min, 5000× *g*) and culture supernatant was used directly (from YEPD cultivation) or after purification (from YMM-glucose-NaNO_3_ cultivation) for activity and hydrolysis experiments.

Purification of ACut2-6hp was done by Ni-NTA-agarose (Novagen, Darmstadt, Germany) under native conditions with imidazole for elution, as recommended by the manufacturer. Three elution steps were done, each with 2.5 mL of elution buffer. After elution, these fractions were desalted with PD10 columns (GE Healthcare, Freiburg, Germany) to remove imidazole [40].

### 2.3. Assay for Determination of ACut2-6hp Activity

Assay was performed according to [42]. A concentration of 0.6 mM pNP-butyrate was used as substrate in 100 µL 50 mM sodium phosphate buffer, pH 7.5. Reactions were started by the addition of the substrate-buffer mix to the culture supernatant, and were measured for 4 min at 405 nm. The control was a substrate-enzyme mix with additional buffer instead of enzyme. The change in the absorbance of the control reaction was subtracted from that of the experimental reaction giving dA, which was then transformed to the pNP concentration via the Beer–Lambert equation (A = ε·c·d, where A = absorbance, b = path length (cm), c = concentration (M), and ε = 18,100 M^−1^cm^−1^ for pNP).

One unit (1 U) of enzyme activity was defined as the amount of enzyme required to hydrolyze 1 µmol pNP-butyrate to pNP per min at 30 °C, pH 7.5.

### 2.4. DEA Hydrolysis by ACut2-6hp

DEA hydrolysis was performed in a volume of 200 µL in 100 mM buffers (sodium acetate buffer, pH 4 and 5; sodium phosphate buffer, pH 5 to 7.5; TRIS-HCl, pH 8 to 9). Enzyme or immobilized enzyme and buffers were given to 2 mL reaction tubes and reactions were started by the addition of 2 µL pure DEA, so that the final DEA concentration was 50 mM. It was incubated at 30 °C, if not mentioned otherwise. At various times DEA, MEA and AA were extracted from the solution.

### 2.5. DEA, MEA and AA Extraction from the Solution and Analysis

Volumes of 200 µL 500 mM TRIS-HCl pH 8 and 400 µL methyl tert-butyl ether (MTBE) were added to a 200 µL DEA hydrolysis reaction. After extraction, 200 µL MTBE phase was transferred to a new tube. A second extraction with further 200 µL MTBE was done after acidification to pH 2 with 50 µL 1 M HCl. Again, 200 µL were transferred to the new tube, and from that mixture 100 µL were evaporated at room temperature. Then, 50 µL dry acetonitrile were added to the samples, and 6.25 µL 1:100 diluted 2-nonanone in dry acetonirtrile (ACN) were added as the internal standard.

Analysis was done via GC-FID measurement with an Agilent DB-5MS, L × I.D. 30 m × 0.25 mm, df 0.25 μm column. After 1 min at 80 °C, the temperature was ramped up to 120 °C at 30 °C min^−1^, then the temperature was increased more slowly, at 10 °C min^−1^, up to 200 °C. The detection temperature was 220 °C.

### 2.6. Determination of the Kinetic Constants of ACut2-6hp Supernatant to DEA and MEA 

Determination of the Michaelis–Menten constant K_M_ was done with desalted ACut2-6hp supernatant by PD10-columns (GE Healthcare, Freiburg, Germany).

The hydrolysis of DEA to MEA or MEA to AA was followed by an indirect assay using a coupled reaction with pH indicators [43]. In the assay, 2 mM sodium phosphate buffer pH 7.2 and 0.5 mM pNP or sodium acetate buffer pH 4.9 and 0.0125 mM bromcresol green were used. 

Two solutions were prepared, containing buffer and indicator with or without the substrates DEA or MEA. For MEA stock solution in buffer/indicator, NaOH was added until the corresponding pH was reached and the absorption of the solution at 405 nm (pNP) or 620 nm (bromcresol green) was identical to the solution without MEA. Reactions were started by the addition of ACut2-6hp preparation, and time-dependent measurement was done at 405 nm for pNP or 620 nm for bromcresol green. A reaction without enzyme was carried along as a control.

For evaluation, dA was plotted against substrate concentration as a v-[S]-plot, and Hanes linearisation was performed, confirming the hyperbolic trend.

### 2.7. Immobilization of ACut2-6hp on Purolite Carriers

Purolite carriers ECR1030M (divinylbenzene and methacrylate) for adsorption, as well as ECR8285 (epoxy/butyl methacrylate) for covalent bonding of the enzyme to a carrier, were preequilibrated with 10 mM buffer. After suction over a filter, enzyme preparations were added to the carriers. Immobilization was done at room temperature by slow rotation of the mixtures for 24 h. Carriers were sucked, washed with 10 mM buffer, and the used for hydrolysis reactions.

### 2.8. Up-Scaling of DEA Hydrolysis

DEA hydrolysis with ACut2-6hp culture supernatant was done in a volume of 44 mL. An enzyme activity of 20 U was used in 40 mL 100 mM sodium phosphate buffer pH 7.5. 

For immobilized ACut2-6hp, 20 U were bonded to 1 g ECR1030M or ECR8285 as described above, and 40 mL 100 mM sodium phosphate buffer pH 7.5 were added.

The mixture was stirred and pH was set to 7.5 on the titrator, before the reaction was started by the addition of 4 mL DEA (453 mM). The pH of the reaction mixture was kept constant by titration with 5 M NaOH. Incubation was done at room temperature for one or two days.

Products were isolated by two extraction steps with 40 mL MTBE. First, DEA was extracted at pH 7.5 and, after adjusting the pH to 2 with 12 M HCl, MEA and AA were isolated. Solvent was distilled off and fractions were analyzed via GC-FID, as described in Section 2.5.

## 3. Results

### 3.1. DEA Hydrolysis by Culture Supernatant of B. raffinosifermentans Strain G1212/YRC102-ACut2-6H

**Figure 2:**
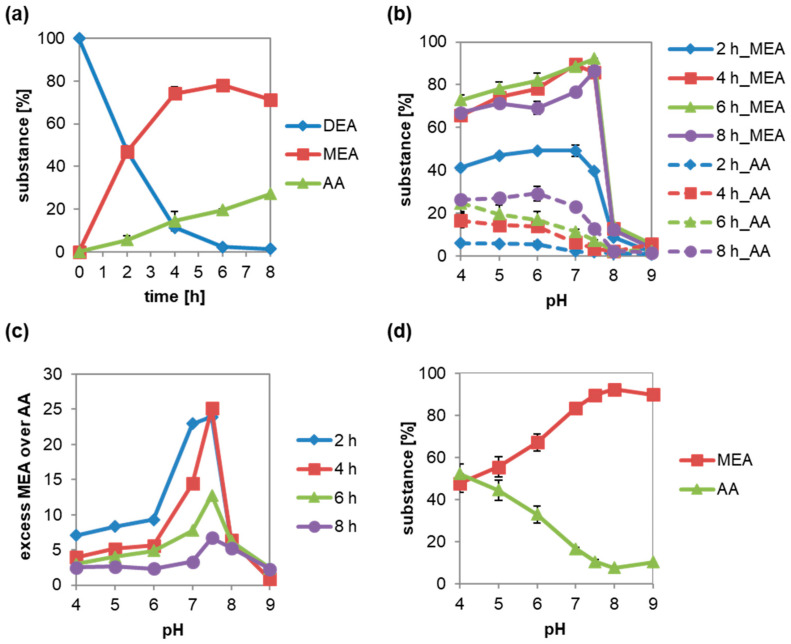
Diethyl adipate (DEA) hydrolysis catalyzed by ACut2-6hp depending on pH. Hydrolysis was done in a volume of 200 µL with 50 mM DEA (**a**–**c**) or monoethyl adipate (MEA) (**d**) in 100 mM sodium acetate pH 5 (**a**) or pH 4–5, 100 mM sodium phosphate pH 6–7.5, or 100 mM TRIS-HCl pH 8–9 with 0.6 µg or 0.015 U ACut2-6hp (**b**–**d**). (**a**) MEA and adipic acid (AA) product concentrations, as well as (**b**) excess AA over MEA, were determined. After 2, 4, 6 and 8 h of incubation, DEA, MEA, and AA levels were determined. (**c**) MEA and AA concentrations after 18 h. Measurements were made in duplicate. Represented are the mean ± SD for the diagrams (**a**,**b**,**d**).

A concentration of 50 mM DEA was hydrolyzed by the addition of 5 µL culture supernatant of the strain G1212/YRC102-ACut2-6H (0.015 U ACut2-6hp activity, 0.6 µg protein) in 100 mM sodium acetate buffer pH 5.0 in a reaction volume of 200 µL (Figure 2a).

In 6 h, DEA was nearly completely hydrolyzed. Monoester MEA formed continuously for 4 h with nearly 20% h^−1^, and the concentration stayed nearly constant from 4 to 6 h at around 76%, and then decreased slowly. AA was synthesized nearly six times slower, with 3.5% h^−1^ over the whole time up to a concentration of 27%. The maximum MEA concentration formed was 78% with 19% AA being present.

The reason for the low selectivity of ACut2-6hp culture supernatant cannot be explained by impurities of the enzyme preparation with two other cutinases. DEA hydrolysis catalyzed by purified ACut2-6hp was non-selective for monoester synthesis as well (data not shown).

### 3.2. Diacid Reduction at DEA Hydrolysis by Culture Supernatant Containing Cutinase ACut2-6hp from B. raffinosifermentans

The pH value during the reaction was varied to increase ACut2-6hp specificity for DEA monohydrolysis by culture supernatant (Figure 2b,c). In the tested pH range from 4 to 9, reaction took place up to pH 7.5 (Figure 2b), in which between 39 and 49% MEA was synthesized in 2 h. This means that the reaction velocity of MEA synthesis stayed constant, independent of the pH. AA concentration was below 6%. The highest MEA yield of 92% was formed at pH 7.5 after 6 h. Lowering the pH and elongating the incubation time decreased the monoester yield due to its hydrolysis to AA. At pH 7.5, the excess of MEA over AA was highest during the whole time (Figure 2c). After 6 h, up to 92% MEA and 7% diacid were present.

However, pH optimization increased the yield of MEA by 14%, from 78% to 92%, whereas AA decreased by 12%, from 19% to 7%.

MEA hydrolyzed only at a pH lower than 8 by ACut2-6hp culture supernatant, which confirms previous results (Figure 2d). The highest AA concentration of 42% was formed at pH 4, decreasing slowly to 2% with the increase to pH 7.5. The inhibition of MEA hydrolysis at pH 7.5 explains the high MEA yields at pH 7.5 if DEA is hydrolyzed.

**Figure 3:**
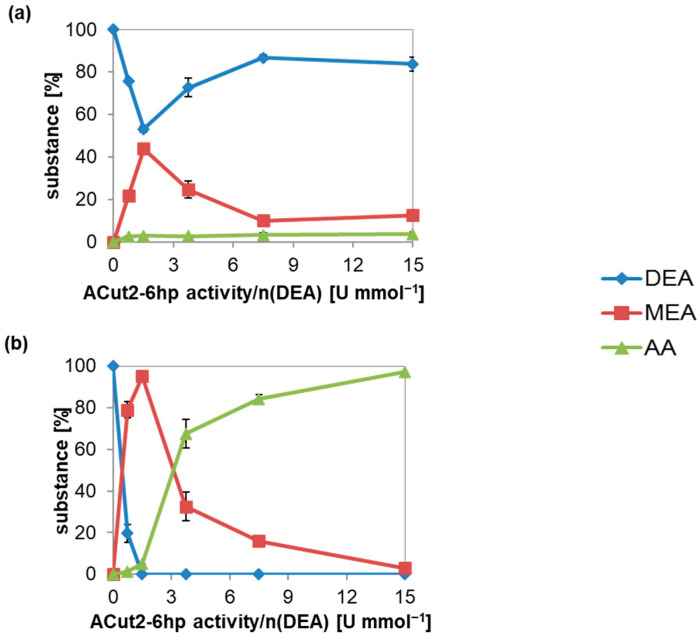
DEA hydrolysis catalyzed by ACut2-6hp depending on the enzyme concentration. Hydrolysis was done in a volume of 200 µL with 50 mM DEA in 100 mM sodium phosphate pH 7.5 with different enzyme concentrations for 2 (**a**) or 16 h (**b**) by ACut2-6hp containing culture supernatant. An ACut2-6hp activity/n(DEA) of 2.5 U mmol^−1^ corresponds to 5 µL Acut2-6hp culture supernatant, or 0.02 U, or 0.57 µg protein. Measurements were made in duplicate. Represented are the mean ± SD.

Additionally, enzyme concentration determines reaction specificity (Figure 3a). When enzyme activity increased two-fold from 0.75 to 1.5 U mmol^−1^, DEA hydrolysis accelerated proportionally from 22 to 44% MEA formed in 2 h. The further increase of the enzyme concentration to 3.75 U mmol^−1^ and even higher (up to 15 U mmol^−1^) decreased the MEA concentration to 10% per 2 h. AA concentration was lower than 4% for all tested enzyme concentrations.

Although the DEA hydrolysis was fastest with 1.5 U mmol^−1^ ACut2-6hp, only minimal AA was found after 16 h (Figure 3b); up to 95% MEA and only 4.9% AA were synthesized. Higher enzyme activities promoted the synthesis of the unwanted diacid AA, which was formed up to 97% in 16 h when 15 U mmol^−1^ cutinase activity was used.

The same dependency of enzyme activity to monoester selectivity was found for purified ACut2-6hp (data not shown).

### 3.3. Kinetic Constant K_M_ of ACut2-6hp Culture Supernatant for the Hydrolysis of DEA and MEA

The Michaelis–Menten constant K_M_ was determined for DEA and MEA as substrates with desalted ACut2-6hp supernatant (Table 2). The K_M_ value for DEA at pH 7.2 could not be determined, because in the range of the DEA solubility up to 15 mM, reaction velocity was still increasing linearly.

Nonetheless, it could be proven that the K_M_ value was more than 10 times lower for MEA compared to DEA at pH 7.2 (data not shown). ACut2-6hp affinity was eight times higher to MEA compared to DEA at pH 4.9. Therefore, the specificity of ACut2-6hp for the DEA monohydrolysis is higher at pH 7.5 compared to pH 4.9.

### 3.4. ACut2-6hp Immobilization by Adsorption and Covalent Bonding to Carriers

Purified ACut2-6hp was adsorbed to copolymer of divinylbenzene methacrylate, as well as covalently bonded to epoxy/butyl methacrylate in 10 mM sodium acetate buffer pH 5 with yields over 99% for both variants concerning pNP-butyrate hydrolysis.

Immobilization of purified ACut2-6hp changed the biochemical characteristics (Table 3).

Immobilized enzymes could be reused for more than 50 or 20 cycles, so there was no enzyme detachment. 

In addition, culture supernatant can be used for the immobilization with yields of 99.7% or 97.5% for adsorption or covalent bonding concerning pNP-butyrate hydrolysis. 

DEA was hydrolyzed unselectively (data not shown). That is the reason why the immobilization procedure and reaction conditions were optimized.

### 3.5. Decreased Diacid Formation at DEA Hydrolysis by Immobilized ACut2-6hp Culture Supernatant

Diacid formation should be reduced by optimizing the pH during immobilization, as well as the pH and temperature during the reaction.

**Figure 4:**
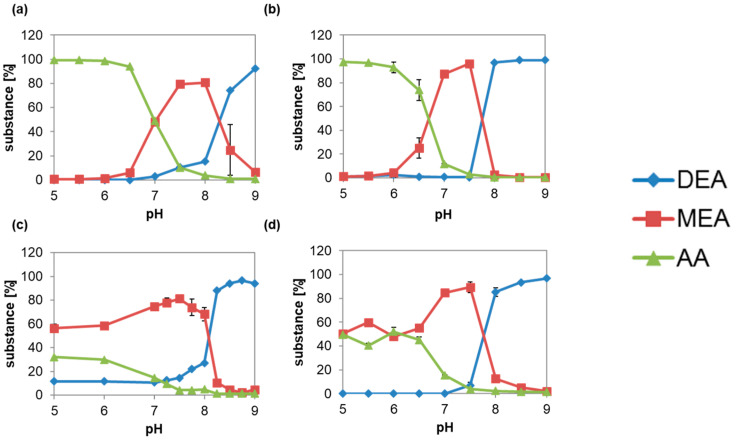
Minimization of AA formation in DEA hydrolysis catalyzed by ACut2-6hp adsorbed (**a**,**c**) or covalently bonded (**b**,**d**) in dependence of pH during immobilization (**a**,**b**) and pH during reaction (**c**,**d**). Immobilizates were loaded with 4 mg protein g^−1^ carrier. Measurements were made in duplicate. Represented are the mean ± SD.

Immobilization at pH 5 to 9 in sodium phosphate buffer pH 7.5 at 30 °C was chosen for hydrolysis (Figure 4a,b). Incubation at pH 5 to 6.5 for adsorption or pH 5 to 6 for covalent bonding gave nearly 100% AA. Afterwards, MEA concentration increased up to 80% at pH 7.5–8 with 4% AA, or 96% at pH 7.5 with 3% AA. DEA is only slightly hydrolyzed if pH is over 8.5 or 8.

When immobilization was done at pH 7.5, MEA concentration increased to 60% for the reaction at pH 5 to 6 or 6.5 for both immobilization methods (Figure 4c,d). Maximum MEA was formed at pH 7.5, and decreased below 10% over pH 8 or from pH 8 for adsorption or covalent bonding.

**Figure 5:**
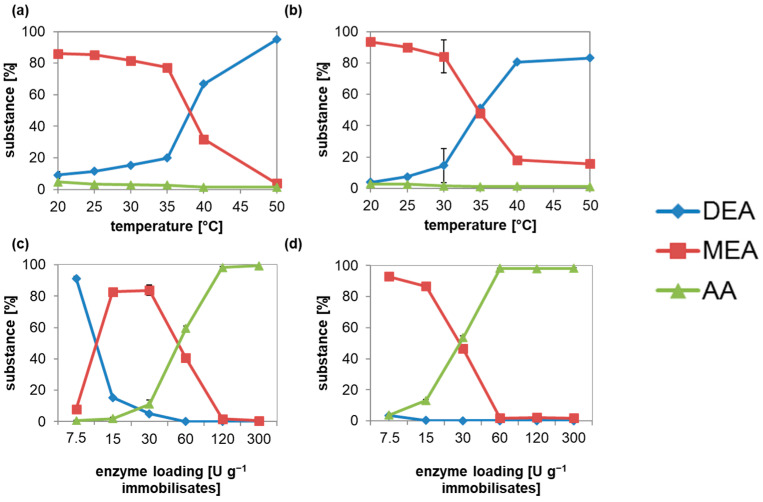
Minimization of AA formation in DEA hydrolysis catalyzed by ACut2-6hp adsorbed (**a**,**c**) or covalently bonded (**b**,**d**) in dependence of temperature during reaction (**a**,**b**) and enzyme loading (**c**,**d**). The activity of 7.5 U g^−1^ corresponds to 1 mg protein g^−1^ carrier of ACut2-6hp. Measurements were made in duplicate. Represented are the mean ± SD.

Interestingly, 10% more AA was synthesized with covalently bonded ACut2-6hp at pH 5–6.5, with 40%. The influence of the reaction temperature on the selectivity was relatively low after optimization of the prior characteristics (Figure 5a,b). Reactions with adsorbed and covalently bonded Acut2-6hp slowed down from 86 or 93% MEA to 77 or 84% per day with the increase of the reaction temperature from 20 to 35 or 30 °C, and then decreased drastically to the minimum of 4 or 18% MEA per day at 50 or 40 °C. This result confirms the higher temperature stability of the adsorbed compared to the covalently bonded enzyme.

Enzyme loading of the immobilizates had a major effect on the selectivity of the DEA hydrolysis (Figure 5c,d). Only 15 to 30 U ACut2-6hp g^−1^ carrier obtained over 80% MEA yield for adsorption, and even lower loadings of 7.5 U g^−1^ offered a maximum MEA concentration of 93% for covalent immobilization. When higher activities were bonded on the carriers, MEA was further hydrolyzed to AA, which increased to 100% for enzyme loadings of 120 or 60 U g^−1^ carrier or higher.

Altogether, immobilizations as well as DEA hydrolysis reactions should be done at pH 7.5, between 20 to 30 °C, with enzyme loadings of 15–30 (adsorption) or 7.5–15 U g^−1^ carrier (covalent bonding). Under these conditions, immobilization was successful with yields of 94.3% for adsorption and 89.0% for covalent bonding. Efficiency and activity recovery were in both cases over 100%, because activity was higher for immobilizates compared to the culture supernatant (data not shown). Thus, there has to be an activation of the biocatalyst ACut2-6hp by immobilization.

### 3.6. Up-Scaling of DEA Hydrolysis by Culture Supernatant Containing Cutinase ACut2-6hp or Immobilized Cutinase ACut2-6hp from B. raffinosifermentans at Constant pH

After optimization of DEA hydrolysis by different ACut2-6hp variants, it was scaled up to a volume of 40 mL. DEA concentration was increased eight times to 450 mM.

Culture supernatant containing cutinase ACut2-6hp was used with 1 U mmol^−1^ DEA (10 mL culture supernatant, 20 U, 1 µg protein). After one day of incubation, 3.4 g product was isolated by extraction of the whole batch (Table 4). There were 84.0% isolated yield with 7.3% DEA, 86.5% MEA and 6.2% AA. Extraction was done in two steps: first at pH 7.5 for isolation of DEA, giving 0.3 g with 84.4% DEA and 15.6% MEA, and second after acidification to pH 2, which gave 3.1 g product with 93.3% MEA and 6.7% AA.

When incubation was prolonged to two days, 3.2 g product was isolated with 87.2% MEA and 12.8% AA. Thus, isolated MEA yield dropped 3% to 79.7% compared to the shorter incubation time with a 6.1% lower MEA concentration.

Results from the 200 µL batches could be confirmed; nevertheless, further increase of selectivity to MEA synthesis is necessary to make the process industrially applicable.

Adsorbed ACut2-6hp was used with an activity of 20 U (10 mL or 1 µg protein) loaded to 1 g carrier (Table 4). After 22 h, the solution was clear and 3.1 g product was extracted containing 98% MEA and 2% AA. Isolated MEA yield was 87.8% and a space-time yield (STY) of 2.6 g L^−1^h^−1^ was achieved. After the first reaction cycle, the immobilized enzymes were reused for a second reaction cycle with a five times higher DEA concentration of 1.7 M. Five days after incubation, 15.6 g of 92.3% MEA was isolated. A STY of 1.5 g L^−1^h^−1^ was reached, which is more than 40% lower compared to the first batch with the lower substrate concentration.

Covalently bonded crude ACut2-6hp gave, under the same conditions, 2.8 g of 98% monoester and 2% diacid after 48 h incubation for 450 mM DEA concentration. This means that MEA yield decreased to 77.3% and STY to 1.12 g L^−1^h^−1^. In addition, product was unclear due to carrier dust, and there was an unknown peak found in the GC-FID measurement.

In summary, reactions with immobilized enzyme preparations gave a 5% higher product concentration with 11.5% more MEA inside, and thus they were more selective compared to that of the free enzyme. Covalently bonded ACut2-6hp is less attractive for MEA synthesis compared to the adsorbed preparation, although it seems to be the more selective enzyme preparation after optimization in the small scale. Adsorbed ACut2-6hp can be bonded with doubled loading capacity to the carrier, gave higher yields with up to 98% purity for MEA, and could be reused with an even higher substrate concentration and two-fold STY.

### 3.7. Determination of Substrate Spectrum of ACut2-6hp Culture Supernatant

Various dicarboxylic acid esters and diol diesters were hydrolyzed by ACut2-6hp culture supernatant under optimized conditions (Table 5). The highest activity was found for DEA hydrolysis, and it was assigned 100%. If the side-chain shortens or extends, activity decreases to 7% (dimethyl adipate) or 27% (diisopropyl adipate). Substrates with a shorter main-chain length were hardly hydrolyzed by ACut2-6hp, while a longer main-chain reduced diacid hydrolysis drastically to 68% for eight C-atoms, and then to around 35% for sebacate esters. Diol diesters were not hydrolyzed at all by the biocatalyst.

When incubation time was prolonged, high monoester concentrations over 90% were obtained for diethyl adipate, diisopropyl adipate, dimethyl suberate, dimethylsebacate, and diethylsebacate.

## 4. Discussion

In this communication, the enzyme cutinase ACut2-6hp from the non-conventional yeast *B. raffinosifermentans* was used for the selective hydrolysis of the aliphatic dicarboxylic acid ester DEA to the monoester MEA. Non-optimized ACut2-6hp catalyzed DEA hydrolysis gave only MEA concentrations up to 78% because MEA was further hydrolyzed to the diacid AA (19%). This problem was overcome by various optimizations, gaining high monoester yields up to 98%.

The DEA hydrolysis by ACut2-6hp was only slightly affected by the pH value. Activity was highest at pH 7 and ranges from 84 to 81% at pH 4 to 7.5, which is in contrast to pNP-butyrate hydrolysis activity being at the maximum at pH 5, ranging from 47 to 67% at pH 4 to 6.5 [40]. The pH optimum is comparable to the *B. raffinosifermentans* intracellular lipase Alip2, catalyzing pNP-butyrate hydrolysis best at pH 7 [27].

MEA hydrolysis is dramatically affected by pH increase, with an activity loss of 30% from pH 5 to 6 and 6 to 7, which resembles the pNP-butyrate hydrolysis [40]. This pH dependent activity difference makes it possible to hydrolyze the DEA selectively to MEA, gaining up to 95% chemical yield by the free enzyme at a pH value of 7.5. Validation of the K_M_-values for DEA and MEA hydrolysis at pH 4.9 and 7.2 confirmed the lower affinity of ACut2-6hp to the substrate MEA with the increase of the pH. 

In addition, enzyme concentration influences the reaction. Surprisingly, the highest reaction velocity and selectivity were achieved at the identical enzyme concentration.

As immobilization on different supports influences different enzyme characteristics [44], and even the enantiomeric excess (ee) of asymmetrically hydrolyzed prochiral diesters as for *Rhizopus oryzae* lipase immobilized on Lewatit CNP 105 [26], the effect of ACut2-6hp binding on carriers by adsorption and covalent bonding was tested. The pH and enzyme concentration determines whether the monoester MEA is further hydrolyzed to AA, as previously shown for the soluble enzyme. Independent of the immobilization method, MEA could be selectively synthesized if pH 7.5 was used at the immobilization procedure itself and for the reaction with enzyme loadings of 7.5 or 15 U g^−1^ immobilizates. 

Improving monoester yield by immobilization can be associated with changes in the enzyme structure in the case of lipases immobilized on hydrophobic supports via interfacial activation [45,46]. In addition, the microenvironment of the immobilized enzyme is crucial [47]. ACut2-6hp immobilized on hydrophobic supports has higher accessibility for a hydrophobic substrates as DEA compared to the more hydrophilic MEA. This partition reduces product hydrolysis and increases the maximum MEA yield [48].

Guo et al. [24] minimized diacid concentration in dimethyl bicyclo [2.2.1]heptane-1,4-dicarboxylate hydrolysis by immobilized *C. antarcitca* lipase B in the same way. Lowering enzyme loadings and maintaining the pH close to 7 decreased the monoacid concentration from 1 to 0.6%.

In the literature, there is much written about enantioselective desymmetrization of prochiral diesters by lipases to enantiopure products [24,25,29,48,49,50]. A whole set of biocatalysts was screened, gaining a lipase with an already high monoester yield and ee [25,49]. After, further optimizations were performed. There was nothing written about formed diacids.

This publication focused the desymmetrization of the even more simple non-prochiral aliphatic diester DEA. The reaction conditions for MEA synthesis from DEA were optimized to make the unselective ACut2-6hp selective. The monoester yield depends on pH and enzyme concentration, so that the influence of the incubation time was minimized.

Finally, a chemical yield of 98% MEA was obtained in the present publication, which is higher compared to alternative methods.

DEA saponification with NaOH under reactive distillation for the removal of the monoester gave only a 12% lower yield with 86% MEA [50]. Babler [51] esterified adipic acid with ethanol in water, acidified with sulphuric acid and extracted with benzene, obtaining up to 87.5% yield. The monoester to diester ratio was 96:4, which is only half compared to the isolated product by ACut2-6hp DEA hydrolysis, being 98:2. Transesterification by strongly basic ionic exchanger resins in ester-hydrocarbon mixtures gave 85 to 95% monoester yield, which is still below the results showed here. Alternative monoesters as monoethyl or monomethyl malonate from the corresponding diesters were obtained with 81 to 82% yield with tetrahydrofuran or acetonitrile and aqueous NaOH or KOH by Niwayama and Cho [52]. Further reprocessing of the reaction product gave a nearly 100% purity for the half-esters.

However, enzymatic methods were rarely used. Only van Nuland et al. [53] used a cell-based reaction system with acyl-CoA ligase and alcohol acyltransferase in combination with the ω-oxidation pathway of *Pseudomonas putida* for monoester synthesis from fatty acids from C6 to C12. Further experiments have to be conducted to increase the molar yield, which is, at 0.75, relatively low.

Nietz et al. [27] describes the biosynthesis of MEA by an intracellular lipase from *B. raffinosifermentans*, obtaining 96% MEA and 1.6% AA. Up to now, the reaction was not up-scaled. In addition, the enzyme was intracellular and had to be purified before use for DEA hydrolysis. This is cost- and time-consuming. The extracellular ACut2 containing supernatant was directly bonded to the support without further purification steps and gave up to 98% MEA with 2% AA in an up-scaled reaction. The DEA concentration was approximately 50 times higher compared to Ref. [27].

These high MEA yields establish ACut2-6hp supernatant hydrolysis of diesters as an alternative method to existing methods for the production of aliphatic monoesters.

## 5. Conclusions

Altogether, we here show that by optimizing reaction parameters, the unspecific ACut2-6hp can be used for the desymmetrization of DEA and other diesters with high monoester yields of up to 98%.

The supernatant of ACut2 overexpression strain catalyzes the desymmetrization of aliphatic diesters chemoselective at pH 7.5 using an enzyme activity of 1.5 U mmol^−1^ substrate. Under these conditions, 86.5% MEA with 6.2% AA and 7.3% DEA was synthesized and isolated in an up-scaled reaction. Even higher chemoselectivity, gaining 98% chemical pure MEA with only 2% AA, was reached after immobilization of the supernatant on carriers. Essential for this were optimizations of the pH, temperature and enzyme loading. Other diesters were also hydrolyzed chemoselectively under these conditions. Because no enzyme purification is necessary, time and costs are low. Compared to existing methods, yield is higher and the isolation methods are quite simple, making this enzymatic process with the available and efficient Acu2-6hp supernatant interesting for the production of aliphatic monoesters.

For industrial application, a continuous reaction procedure should be developed to further reduce costs and time. The immobilized enzyme could be used in repeated batches, or new substrate could be added after the first reaction cycle while the product solution is removed in correspondence to NaOH addition. In addition, further diester hydrolysis reactions have to be tested in the up-scaling process to confirm the capability of ACut2 supernatant as a new biocatalyst for the production of monoesters.

## Figures and Tables

**Table 1:** Substances produced from monoesters.

	Structure	Name and Function	Literature
1	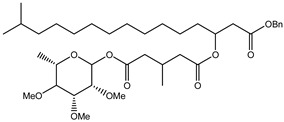	Building block for the synthesis of lipo-nucleoside antibiotic caprazamycin	[14]
2	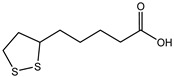	Lipoic acid: natural antioxidant and cofactor of several enzymes	[15]
3	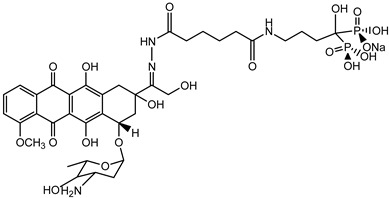	Alendronate-monoethyl adipate-(hydrazone)-doxorubicin: anti-tumor agent for active delivery of doxorubicin to bone metastasis	[16]
4	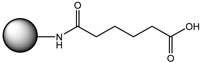	Acid functionalized polystyrene resin for use in solid phase chemistry	[17]

**Table 2:** Michelis–Menten constants of ACut2-6hp to DEA and MEA at pH 4.9 and 7.2.

Substrate	pH	Indicator	Tested (Substrate) Range	K_M_ [mM]	R^2^ for Regression Graph of the v-[S]-Plot
DEA	7.2	para-nitrophenol (pNP)	0–15 mM	n.d.	0.99
DEA	4.9	bromcresol green	0–15 mM	4.98	0.95
MEA	7.2	pNP	0–150 mM	67.69	0.99
MEA	4.9	bromcresol green	0–13 mM	0.61	0.96

n.d., not defined.

**Table 3:** Biochemical characteristics of ACut2-6hp as free, adsorbed, and covalently bound enzyme preparation.

Parameter	Free Enzyme *	Adsorbed Enzyme	Covalently Bound Enzyme
Yield	-	99.2	97.5
pH optimum	5.0 (4.0–6.0)	6.5 (6.0–6.5)	6.0 (5.5–6.0)
Temperature optimum	30–45 °C	55 °C 50–55 °C	50 °C 45–55 °C
Temperature stability	Not stable	30 °C	30 °C
Cycles for re-use	-	>50	>20

* Reprinted/adapted with permission from Ref. [40]. 2022, G. Kunze.

**Table 4:** Isolated product from up-scaled DEA hydrolysis reactions by ACut2-6hp preparations.

	Free Enzyme (40 mL Buffer + 4 mL DEA)	Adsorbed Enzyme (40 mL Buffer + 4 mL DEA)	Adsorbed Enzyme (40 mL Buffer + 20 mL DEA)	Covalently Bound Enzyme (40 mL Buffer + 4 mL DEA)
Product (g)	3.4	3.1	15.6	2.8
Product analysis	7.3% DEA, 86.5% MEA, 6.2% AA	98% MEA, 2% AA	5.7% DEA, 92.3% MEA, 2% AA	98% MEA, 2% AA
Isolated yield MEA (%)	84	87.8	89.7	77.3
Incubation time (h)	24	22	120	48
Space-time yield (STY) (g L^−1^ h^−1^)	2.45	2.6	1.5	1.12

**Table 5:** Substrate spectrum of purified free ACut2-6hp.

Substrate	Monoester in % after 2 h Incubation	Monoester/Diacid in % after 24 h Incubation
Dicarboxylic acid ester		
Diethyl malonate (3C + 2C)	0	0/0
Diethyl succinate (4C + 2C)	4	28.5/0
Dimethyl adipate (6C + 1C)	3	9.6/0
Diethyl adipate (6C + 2C)	42	99.1/0.9
Diisopropyl adipate (6C + 3C)	11	96.0/0
Dimethyl suberate (8C + 1C)	29	93.3/1.8
Dimethyl sebacate (10C + 1C)	15	74.8/3.7
Diethyl sebacate (10C + 2C)	16	42.5/10.3
Diol diester		
1,4-Diacetoxy butane (4C + 2C)	0	0/0
1,6-Diacetoxyhexane (6C + 2C)	0	0/0
1,10-Diacetoxy decane (10C + 2C)	0	0/0

## Data Availability

The data that support the findings of this study are available from the author Marion Rauter upon reasonable request.

## References

[1] Jin X., Li M., Yin L., Zhou J., Zhang Z., Lv H. (2017). Tyroservatide-TPGS-paclitaxel liposomes: Tyroservatide as a targeting ligand for improving breast cancer treatment. Nanomed. Nanotechnol. Biol. Med..

[2] Li W., Hao J., Xiao Y. (2013). Synthesis and in vitro antitumor activities of lupeol dicarboxylic acid monoester derivatives. Arch. Pharm. Res..

[3] Erythropel H.C., Brown T., Maric M., Nicell J.A., Cooper D.G., Leask R.L. (2015). Designing greener plasticizers: Effects of alkyl chain length and branching on the biodegradation of maleate based plasticizers. Chemosphere.

[4] Korsager S., Nielsen D.U., Taaning R.H., Skrydstrup T. (2013). Access to β-Keto Esters by Palladium-Catalyzed Carbonylative Coupling of Aryl Halides with Monoester Potassium Malonates. Angew. Chem. Int. Ed..

[5] Schuster L., Rothmund L., He X., Van Landuyt K.L., Schweikl H., Hellwig E., Carell T., Hickel R., Reichl F.X., Högg C. (2015). Effect of Opalescence^®^ bleaching gels on the elution of dental composite components. Dent. Mater..

[6] Yadav A., Mathur R., Samim M., Lomash V., Kushwaha P., Pathak U., Babbar A., Flora S., Mishra A., Parshad Kaushik M. (2014). Nanoencapsulation of DMSA monoester for better therapeutic efficacy of the chelating agent against arsenic toxicity. Nanomedicine.

[7] Rajagopalan A., Kroutil W. (2011). Biocatalytic reactions: Selected highlights. Mater. Today.

[8] Cabrera Z., Palomo J.M., Fernandez-Lorente G., Fernandez-Lafuente R., Guisan J.M. (2007). Partial and enantioselective hydrolysis of diethyl phenylmalonate by immobilized preparations of lipase from Thermomyces lanuginose. Enzym. Microb. Technol..

[9] Tan J.N., Dou Y. (2020). Deep eutectic solvents for biocatalytic transformations: Focused lipase-catalyzed organic reactions. Appl. Microbiol. Biotechnol..

[10] Aldabbagh F., Katritzky A.R., Taylor R.J.K. (2005). Acid halides. Comprehensive Organic Functional Group Transformations II.

[11] Zhang X., Breslav M., Grimm J., Guan K., Huang A., Liu F., Maryanoff C.A., Palmer D., Patel M., Qian Y. (2002). A new procedure for preparation of carboxylic acid hydrazides. J. Org. Chem..

[12] Lanigan R.M., Starkov P., Sheppard T.D. (2013). Direct Synthesis of Amides from Carboxylic Acids and Amines Using B(OCH_2_CF_3_)_3_. J. Org. Chem..

[13] Matsumoto K., Yanagi R., Oe Y., Badea G.-I., Radu G.L. (2018). Recent advances in the synthesis of carboxylic acid esters. Carboxylic Acid-Key Role in Life Sciences.

[14] Gopinath P., Watanabe T., Shibasaki M. (2012). Studies on Catalytic Enantioselective Total Synthesis of Caprazamycin B: Construction of the Western Zone. J. Org. Chem..

[15] Acker D.S., Park B. (1957). Process of Preparing Alpha-Lipoic Acid Using Dichlorooctanoate and Metal Disulfide. U.S. Patent.

[16] Ye W., Zhao Y., Na R., Li F., Mei Q., Zhao M., Zhou S. (2015). Actively Targeted Delivery of Doxorubicin to Bone Metastases by a pH-Sensitive Conjugation. J. Pharm. Sci..

[17] Stieber F., Waldmann H. (2002). Development of new acid-functionalized resins for combinatorial synthesis on solid supports. Chem. Commun..

[18] Pon R.T., Yu S. (2005). Tandem oligonucleotide synthesis using linker (First BaseTM) phosphoramidites. Nucleic Acids Res..

[19] Vogl O. (1979). Polymers With Functional Groups. Pure Appl. Chem..

[20] Escamilla G.H., Newkome G.R. (1995). Dendritic bolaamphiphiles and related molecules. Advances in Dendritic Macromolecules.

[21] Tejero R., Gutiérrez B., López D., López-Fabal F., Gómez-Garcés J.L., Muñoz-Bonilla A., Fernández-García M. (2018). Tailoring Macromolecular Structure of Cationic Polymers towards Efficient Contact Active Antimicrobial Surfaces. Polymers.

[22] Janović Z., Jukić A., Vogl O. (2010). Spacer groups in macromolecular structures. Polimeri.

[23] Anyanwu U.K., Venkataraman D. (2003). Effect of spacers on the activity of soluble polymer supported catalysts for the asymmetric addition of diethylzinc to aldehydes. Tetrahedron Lett..

[24] Guo Z., Wong M.K.Y., Hickey M.R., Patel B.P., Qian X., Goswami A. (2014). Enzyme-Catalyzed Hydrolysis of Bicycloheptane and Cyclobutene Diesters to Monoesters. Org. Process Res. Dev..

[25] Süss P., Illner S., von Langermann J., Borchert S., Bornscheuer U.T., Wardenga R., Kragl U. (2014). Scale-Up of a Recombinant Pig Liver Esterase-Catalyzed Desymmetrization of Dimethyl Cyclohex-4-ene-cis-1,2-dicarboxylate. Org. Process Res. Dev..

[26] Cabrera Z., Palomo J.M. (2011). Enantioselective desymmetrization of prochiral diesters catalyzed by immobilized Rhizopus oryzae lipase. Tetrahedron Asymm..

[27] Nietz D., Bode R., Kunze G., Rauter M. (2022). A new lipase (Alip2) with high potential for enzymatic hydrolysis of the diester diethyladipate to the monoester monoethyladipate. Enzym. Microb. Technol..

[28] Ehlert J., Kronemann J., Zumbrägel N., Preller M. (2019). Lipase-catalyzed chemoselective ester hydrolysis of biomimetically coupled aryls for the synthesis of unsymmetric biphenyl Esters. Molecules.

[29] Chênevert R., Ngatcha B.T., Rose Y.S., Goupil D. (1998). Regio- and enantioselectivity of the enzyme catalysed hydrolysis of citric acid derivatives. Tetrahedron Asymm..

[30] Torres C., Otero C. (1999). Part I. Enzymatic synthesis of lactate and glycolate esters of fatty alcohols. Enzym. Microb. Technol..

[31] Bianchi D., Bortolo R., Bernardi A., Gagliardi I., Ingrassia A.G.M. (1995). Selective enzymatic hydrolysis of aromatic diesters using esterase from *Brevibacterium imperiale* B222. Biotechnol. Lett..

[32] Kashima Y., Liu J., Takenami S., Niwayama S. (2002). Asymmetric desymmetrization of dialkyl bicyclo[2.2.1]hept-2,5-diene-2,3-dicarboxylates by a thermophilic esterase/lipase. Tetrahedron Asymm..

[33] Goswami A., Kissick T.P. (2009). Enzymatic Desymmetrization of Dimethyl Cylcohex-4-ene-cis-1,2-dicarboxylate to (1S,2R)-2-(Methoxycarbonyl)cyclohex-4-ene-1-carboxylic Acid. Org. Process Res. Dev..

[34] Flors V., Miralles C., Cerezo M., González-Bosch C., García-Agustín P. (2001). Effect of a Novel Chemical Mixture on Senescence Processes and Plant−Fungus Interaction in Solanaceae Plants. J. Agric. Food Chem..

[35] Flors V., Miralles C., González-Bosch C., Carda M., García-Agustín P. (2003). Three novel synthetic amides of adipic acid protect Capsicum anuum plants against the necrotrophic pathogen Alternaria solani. Physiol. Mol. Plant Pathol..

[36] Flors V., Paradís M., García-Andrade J., Cerezo M., González-Bosch C., García-Agustín P. (2007). Tolerant Behavior in Salt-Sensitive Tomato Plants can be Mimicked by Chemical Stimuli. Plant Signal Behav..

[37] Vicedo B., de la O., Leyva M., Flors V., Finiti I., del Amo G., Walters D., Real M.D., García-Agustín P., González-Bosch C. (2006). Control of the phytopathogen Botrytis cinerea using adipic acid monoethyl ester. Arch. Microbiol..

[38] Barros D., Azevedo A.M., Cabral J., Fonseca L.P. (2012). Optimization of Flavor Esters Synthesis by *Fusarium solani pisi* Cutinase. J. Food Biochem..

[39] Baker P.J., Poultney C., Liu Z., Gross R., Montclar J.K. (2012). Identification and comparison of cutinases for synthetic polyester degradation. Appl. Microbiol. Biotechnol..

[40] Bischoff F., Litwińska K., Cordes A., Baronian K., Bode R., Schauer F., Kunze G. (2015). Three New Cutinases from the Yeast Arxula adeninivorans that Are Suitable for Biotechnological Applications. Appl. Environ. Microbiol..

[41] Thomas S., Sanya D.R.A., Fouchard F., Nguyen H.-V., Kunze G., Neuvéglise C., Crutz-Le Coq A.-M. (2019). *Blastobotrys adeninivorans* and *B. raffinosifermentans*, two sibling yeast species which accumulate lipids at elevated temperatures and from diverse sugars. Biotechnol. Biofuels.

[42] Pohanka M. (2019). Biosensors and Bioassays Based on Lipases, Principles and Applications, a Review. Molecules.

[43] Kazlauskas R.J., Reymond J.-L. (2005). Quantitative assay of hydrolases for activity and selectivity using color changes. Enzyme Assays.

[44] Sheldon R., van Pelt S. (2013). Enzyme immobilisation in biocatalysis: Why, what and how. Chem. Soc. Rev..

[45] Rodrigues R.C., Ortiz C., Berenguer-Murcia Á., Torres R., Fernández-Lafuente R. (2013). Modifying enzyme activity and selectivity by immobilization. Chem. Soc. Rev..

[46] Zisis T., Freddolino P.L., Turunen P., van Teeseling M.C.F., Rowan A.E., Blank K.G. (2015). Interfacial Activation of *Candida antarctica* Lipase B: Combined Evidence from Experiment and Simulation. Biochemistry.

[47] Garcia-Galan C., Berenguer-Murcia Á., Fernandez-Lafuente R., Rodrigues R.C. (2011). Potential of Different Enzyme Immobilization Strategies to Improve Enzyme Performance. Adv. Synth. Catal..

[48] Kasche V. (1986). Mechanism and yields in enzyme catalysed equilibrium and kinetically controlled synthesis of β-lactam antibiotics, peptides and other condensation products. Enzym. Microb. Technol..

[49] Sousa H.A., Afonso C.A.M., Crespo J.G. (2000). Kinetic study of the enantioselective hydrolysis of a meso-diester using pig liver esterase. J. Chem. Technol. Biotechnol..

[50] Almilly R.F.K., Alobaidy A.A., Alhassani M.H. (2014). Saponification of Diethyl Adipate with Sodium Hydroxide Using Reactive Distillation. J. Eng..

[51] Babler J.H. (1982). Method of Preparing Monoesters. U.S. Patent.

[52] Niwayama S., Cho H. (2009). Practical large Scale Synthesis of Half-Esters of Malonic Acid. Chem. Pharm. Bull..

[53] van Nuland Y., de Vogel F.A., Eggink G., Weusthuis R. (2017). Expansion of the ω-oxidation system AlkBGTL of Pseudomonas putida GPo1 with AlkJ and AlkH results in exclusive mono-esterified dicarboxylic acid production in *E. coli*. Microb. Biotechnol..

